# Multi-Target In Silico Prediction of Inhibitors for Mitogen-Activated Protein Kinase-Interacting Kinases

**DOI:** 10.3390/biom11111670

**Published:** 2021-11-10

**Authors:** Amit Kumar Halder, M. Natália D. S. Cordeiro

**Affiliations:** 1 LAQV-REQUIMTE/Faculty of Sciences, University of Porto, 4169-007 Porto, Portugal; 2 Dr. B. C. Roy College of Pharmacy and Allied Health Sciences, Dr. Meghnad Saha Sarani, Bidhannagar, Durgapur 713212, India

**Keywords:** MNK-1 and MNK-2 inhibitors, mt-QSAR modeling, virtual screening

## Abstract

The inhibitors of two isoforms of mitogen-activated protein kinase-interacting kinases (i.e., MNK-1 and MNK-2) are implicated in the treatment of a number of diseases including cancer. This work reports, for the first time, a multi-target (or multi-tasking) in silico modeling approach (mt-QSAR) for probing the inhibitory potential of these isoforms against MNKs. Linear and non-linear mt-QSAR classification models were set up from a large dataset of 1892 chemicals tested under a variety of assay conditions, based on the Box–Jenkins moving average approach, along with a range of feature selection algorithms and machine learning tools, out of which the most predictive one (>90% overall accuracy) was used for mechanistic interpretation of the likely inhibition of MNK-1 and MNK-2. Considering that the latter model is suitable for virtual screening of chemical libraries—i.e., commercial, non-commercial and in-house sets, it was made publicly accessible as a ready-to-use FLASK-based application. Additionally, this work employed a focused kinase library for virtual screening using an mt-QSAR model. The virtual hits identified in this process were further filtered by using a similarity search, in silico prediction of drug-likeness, and ADME profiles as well as synthetic accessibility tools. Finally, molecular dynamic simulations were carried out to identify and select the most promising virtual hits. The information gathered from this work can supply important guidelines for the discovery of novel MNK-1/2 inhibitors as potential therapeutic agents.

## 1. Introduction

The mitogen-activated protein (MAP) kinase-interacting kinases, or MNKs, are an important class of kinase enzymes that are activated by extracellular signal-regulated kinases (ERK) as well as p-38 mitogen-activated kinases. Their activation is related to crucial physiological processes such as ribosome assembly and protein synthetic processes [1,2,3]. Two isoforms of MNKs—i.e., MNK-1 and MNK-2, are encoded by two different human genes but both participate in the regulation of transcription that is mediated through the phosphorylation of eukaryotic translation initiation factor 4E (eIF4E) on Ser209 [4]. In fact, MNK-2 is responsible for the basal phosphorylation of elF4E, whereas MNK-1 promotes inducible phosphorylation of eIF4E after activation of MAP kinases. It was also noted that biological substances like Friend leukemia integration 1 specifically regulate the expression of MNK-1 but not MNK-2 [5]. The role of MNKs in various diseases has been established over the last few decades but their main interest stems from developing inhibitors of MNKs as anticancer agents [5,6]. eIF4E was found to be a proto-oncogene which actively participates in mRNA translation, promoting tumor proliferation, invasion and metastasis, whereas its phosphorylation is regulated by multiple oncogenic cell-signaling pathways [5]. MNK-1 and MNK-2 are structurally distinct from other kinases mainly due to the presence of an Asp-Phe-Asp or DFD motif, as well as short inserts in their catalytic pockets [4]. Such a structurally unique motif alters the ATP binding at the catalytic site and, at the same time, renders MNKs as bimolecular targets against which highly specific inhibitors may be designed [3,5,7,8]. The combination of MNK inhibitors with tamoxifen and dasatinib has been reported to improve overall therapeutic outcomes against some cancers. Apart from cancer, MNK-1/2 inhibitors are also implicated as possible biomolecular targets for auto-immune diseases, inflammation and viral infection (e.g., by buffalopox virus) [5]. At the moment, three MNK-1/2 inhibitors—namely, BAY1143269, eFT508 and ETC-206—are under clinical trial for the treatment of solid tumors and leukemia [5,7].

Undoubtedly, there is an urgent need to develop novel potent and selective MNK-1/2 inhibitors. Lately, rational drug design strategies, in particular ligand- and structure-based in silico design strategies, have emerged as promising alternative non-animal approaches for the design of novel lead molecules in a fast and effective manner. To this end, such approaches have already been employed by various research groups for the design of MNK inhibitors, as described in a recently published review by Gagic et al. [9].

This works aims at predicting MNK-1 and MNK-2 inhibitors by validated multi-target or multi-tasking quantitative structure–activity relationship (mt-QSAR) modeling based on the Box–Jenkins approach, which allows one to merge information concerning multiple biological targets and the experimental assay conditions associated with the determination of their endpoints into a single dataset. Evidently, models developed with such kinds of multi-tasking approaches have a broader scope as compared to single target models, in which either only one MNK isoform is considered or the variations coming from different experimental assay conditions are disregarded. To the best of our knowledge, this is the first report on multi-tasking in silico modeling of MNK-1/2 inhibitors. The developed mt-QSAR models were utilized for screening a focused kinase library to obtain the most potential virtual hits, which were further investigated by drug-likeness predictions, similarity search analysis and molecular dynamics (MD) simulations. Furthermore, we made an effort, for the first time, to provide the most predictive mt-QSAR model as a FLASK based web-application (for running in a local machine), mainly to assist future users in applying this model for their own virtual screening-based predictions in a fast and effective manner.

## 2. Materials and Methods

### 2.1. Dataset and Calculation of Molecular Descriptors

The dataset employed and retrieved from the public database ChEMBL (https://www.ebi.ac.uk/chembl/, accessed on 7 June 2021) comprises a total of 1892 compounds with activity estimated against MNKs under different experimental conditions (*c_j_*). The latter are better expressed as an ontology [10,11,12,13] of the form *c_j_* → (*b_t_*, *m_e_*, *a_t_*), that is, by defining them according to the following elements: *b_t_*—the ‘biological target’, accounting for the specific MNK enzyme isoform against which the compounds have been tested, *m_e_*—the kind of ‘measures of biological effects’ considered, namely, half-maximal inhibitory concentration (IC_50_), inhibition (*K_i_*) or dissociation (*K_d_*) constants, and *a_t_*—the ‘assay type’, focusing on either the binding affinity (B) or functional (*F*) responses. As such, each data-point of the input dataset pertains to one specific combination of the elements *b_t_*, *m_e_*, and *a_t_* or experimental condition *c_j_*, and then classified into two categories: positive (IA*c_j_* = +1; for high inhibitory potential) or negative (IA*c_j_* = −1; for low inhibitory potential). We selected a unique cut-off value for all measures of biological effects for classifying the data-points as positives (*m_e_* > 100 nM). The low sub-micromolar cut-off values ensured a more stringent search for potent hits using the mt-QSAR models.

The collected input dataset was then properly curated to remove duplicates, i.e., those data-points that have the same IA*c_j_* value and experimental conditions (details of this input dataset are given in Table S1). Then, the SMILES structures of the compounds were first converted to 2D structures (.sdf format) using the MarvinView software (https://docs.chemaxon.com/display/docs/marvinview.md, accessed on 7 June 2021). Subsequently, these structures were standardized by resorting to the ChemAxon Standardizer tool using the following options: strip salts, aromatize, neutralize and add explicit hydrogen atoms [14]. The starting molecular descriptors were calculated with such standardized structures with the newly launched AlvaDesc.v.0.1 software [15] by employing the OCHEM webserver [16]. For the calculation of 3D descriptors, a geometry optimization of the compound structures was carried out using Corina [17].

As mentioned previously, we applied the Box–Jenkins moving average (BJMA) approach to produce new modified descriptors for each data-point, which includes information about the chemical structure and physicochemical properties of the compounds as well as the experimental conditions employed. The BJMA approach has been thoroughly described in our recent work [18,19,20], and thus, a detailed description is avoided here. Briefly, by using our in-house QSAR-Co-X software [18], the dataset was first divided into a training set and an external validation set using the random division scheme (settings: random seed = 2 and external validation set size = 30%). The BJMA approach was subsequently applied to compute the new modified descriptors (Δ(*D_i_*)*c_j_*) for both such sets using the simplest method— ‘Method1’ of QSAR-Co-X [18], that is represented as follows:

(1) $$\Delta (D_{i})c_{j}=D_{i}-avg(D_{i})c_{j},$$

*D_i_* stands for the input starting descriptors and *avg*(*D_i_*)*c_j_* for their averages—i.e., arithmetic means of active chemicals for a specific element of the ontology *c_j_*.

The training set was then further divided into a sub-training set and a test set using the random scheme (settings: random seed = 2 and test set size = 20). Since the external dataset participates neither in model formation nor in descriptor calculation, it is considered as a ‘true validation set’. The test set, on the other hand, may be used both as a calibration set for selecting the best model and as a test set for validating the model.

### 2.2. Model Development and Evaluation

#### 2.2.1. Linear mt-QSAR Models

To begin with, we attempted to derive linear interpretable models based on the computed modified descriptors by linear discriminant analysis (LDA) using three different feature selection algorithms. Two of these schemes are implemented in QSAR-Co-X and have been discussed earlier [18], i.e., (i) fast stepwise (FS-LDA) and (ii) sequential forward selection (SFS-LDA)-based LDA. The last scheme we employed is the genetic algorithm-based LDA or GA-LDA, which is implemented in our previously launched Java-based tool QSAR-Co [19]. The methodology for developing FS-/SFS-/GA-LDA models has been extensively discussed in our previous works [18,19], thus we will underline here only the most important aspects. For all linear models, an inter-correlation cut-off value of 0.95 and a variance cut-off value of 0.001 were set to discard all highly correlated descriptors and those with less variance. The maximum number of descriptors allowed was 10. In FS-LDA, both *p*-values ‘to enter’ and *p*-values ‘to remove’ were set as 0.05. The SFS-LDA models were developed using two different scoring parameters, i.e., ‘Accuracy’ (overall ratio of correct classifications) and ‘AUROC’ (area under the receiver operating characteristic curve) scores [18]. The GA-LDA models were built with the following parameters, namely: (a) mutation probability = 0.3, (b) initial number of generated equations = 100 and (c) number of equations chosen for each generation = 30. The most predictive GA-LDA model was selected from 20 different runs [20].

#### 2.2.2. Post-Selection Similarity Search-Based Modification

In the ‘Post-selection similarity search-based modification’ (short form PS3M), the generated linear model is treated as a reference model and is then trained with all descriptors of the modeling data-matrix. In doing so, each descriptor of the reference model is used to find the m number (user-specific) of descriptors that have a minimum Euclidean distance (ED) from it. The distance between the two descriptors (*D*_1_ and *D*_2_) is simply calculated as follows:

(2) $$d(D_{1},\;D_{2})=\sum_{i=1}^{n}(D1_{i}-D2_{i}{)}^{2},$$

where *n* is the number of data-points. In this work, the value of *m* was kept fixed as 10.

As such, if the reference model contains *p* number of descriptors, after removing from it descriptors with ED ~ 0, we may expect to obtain an (m*p) number of alternative models that could be referred as ‘*similar*’ linear models. The PS3M assumes that a more predictive linear model may be obtained from these similar alternative models. If any better model is found, it is then employed as the next reference model for the next run and these runs are continued until no better model is reached. For selecting the best classification model, we used the average Matthews correlation coefficient (MCC) value obtained from both the *sub-training* and *test* sets. Nevertheless, it is important to mention that PS3M does not allow compromising of the statistical quality of the sub-training set from which the original model was established. Therefore, if the MCC value of the sub-training set is dropped during this process, an alternative better model is not attempted. All PS3M refinements were performed with our own *in-house* developed tool, which is available at https://github.com/ncordeirfcup/PS3M_v2 (accessed on 18 June 2021).

#### 2.2.3. Non-Linear mt-QSAR Models

Non-linear mt-QSAR models were derived using five different machine learning (ML) tools, namely: (i) *k*-Nearest Neighborhood (*k*NN) [21], (ii) Bernoulli Naïve Bayes (NB) [22], (iii) Support Vector Classifier (SVC) [23], (iv) Random Forests (RF) [24], (v) Gradient Boosting (GB) [25] and (vi) Multi-Layer Perception (MLP) [26]. Two different types of non-linear models were developed with a limited number of descriptors (i.e., 10) and an unlimited number of descriptors. For developing non-linear models with a limited number of descriptors, the features were first selected with the help of IMMAN software (http://mobiosd-hub.com/imman-soft/, accessed on 27 June 2021) that ranks the most discriminating features on the basis of the differential Shannon entropy [27]. Even though all descriptors were considered for developing the second type of non-linear models, a data-pretreatment was carried out using an inter-correlation cut-off of 0.95 and a variance cut-off of 0.001 to remove highly correlated and/or near constant descriptors. All non-linear models were developed with QSAR-Co-X [18], using a 5-fold cross-validation (CV)-based hyper-parameter optimization scheme. Details about the parameters tuned for each of these ML tools were provided and can be seen in our previous work [18]. Additionally, a deep neural network (DNN) model was also built using the Python-based Keras module (https://www.tensorflow.org/api_docs/python/tf/keras, accessed on 27 June 2021). The basic architecture of this DNN is based on six hidden layers with a gradual decrement in the number of neurons. Details on the DNN architecture employed here are given in the Supplementary Materials (Table S2). Note that the same architecture was earlier proposed and presented by Sosnin et al. [28]. A grid search was first carried out with the sub-training set to select the learning rate, batch size and epochs. During this search, a 5-fold CV of the sub-training set was taken into consideration. Subsequently, the DNN model was developed with these selected parameters using the sub-training set and, similar to other ML tools, the external predictively of the model was first determined using the test set and finally with the external validation set. Two Jupyter Notebook files (DNN_MNK_file1.ipynb and DNN_MNK_file2.ipynb) are provided in the Supplementary Materials to demonstrate how these tasks related to DNN were carried out.

#### 2.2.4. Model Evaluation

The models were estimated with several statistical parameters, computed using our *in house* tool QSAR-Co-X [18]. For example, goodness of fit of the linear QSAR models was judged by standard indices such as the Wilk’s lambda (λ), the Fisher’s statistic index (*F*), and the corresponding *p*-values (*p*) [29]. The quality of all derived classification models was further checked by other parameters, such as number of true positives (TP), true negatives (TN), false positives (FP), false negatives (FN), sensitivity (Sn; ratio of correct classification of positives), specificity (Sp; ratio of correct classification of negatives), accuracy (Acc), F1-score, the Matthews correlation coefficient (MCC), and the AUROC score. ROC plots were also automatically generated by the QSAR-Co-X tool for all the mt-QSAR models. In order to check if the linear models are unique in nature, we performed a *Y_c_*-randomization test, which is a modification of the *Y*-randomization test [30]. In the *Y_c_* randomization test, both the dependent parameters and the experimental elements were scrambled 100 times to produce randomized Box–Jenkins modified descriptors for producing new randomized models. The average of the Wilks *λ* values (*λ_r_*) and that of the randomized accuracy (Accuracy*_r_*) were then compared to the corresponding parameters of the original model to check the uniqueness of the latter. Additionally, another parameter named random accuracy ($Acc_{rnd}$) was proposed by Lučić et al. [31,32], and it is calculated using the following formulae:

(3) $$Acc_{rnd}=100*\frac{((\mathrm{TP}+\mathrm{FN})(\mathrm{TP}+\mathrm{FP})+(\mathrm{TN}+\mathrm{FN})(\mathrm{TN}+\mathrm{FP})}{N^{2}}\;,$$

where, *N* is the total number of compounds for a specific set.

Similar to Accuracy*_r_*, $Acc_{rnd}$ is also compared to the original accuracy value of the model and a large difference of the latter with the original accuracy of the model clearly demonstrates that the classification model provides a significant level of useful information over the maximal level of random accuracy [31,32].

### 2.3. Similarity Search Analysis

Similarity searching was used as an important step for further filtering the virtual hits. The similarity search analysis was carried out using our newly developed another *in-house* tool SIMSEARCH (https://github.com/ncordeirfcup/SIMSEARCH, accessed on 9 July 2021), which calculates the Tanimoto similarity [33] between query and target compounds based on various fingerprints. In the current work, extended-connectivity fingerprints with up to four bonds (ECFP4), were used to obtain structurally similar query compounds with respect to the target compounds [34,35].

### 2.4. Molecular Dynamics Simulations

The X-ray crystal structures of MNK-1 (PDB ID: 5WVD [36]) and MNK-2 (PDB ID: 6CK3 [37]) were retrieved from the Protein Data Bank [38]. The compounds were first docked at the catalytic site of these enzymes defined by the location of the small molecule inhibitors complexed with these proteins, using the Autodock 4.2 package [39]. A grid size of 50 Å × 50 Å × 50 Å with a grid-point spacing of 0.375 Å was defined from the bound ligands located at the catalytic site of the proteins. The remaining docking protocols were the same as described in our earlier work [40]. All these docked complexes were subjected to 50 ns molecular dynamics simulations using the Amber 12 MD package [41,42]. To do so, the protonation states of the amino acid residues of each protein were set at pH = 7.0 with the help of the PDB2PQR server (https://server.poissonboltzmann.org/, accessed on 12 July 2021) [43], whereas the ligand parameterization was performed with the Amber’s Leap script using the general AMBER forcefield with the set of auxiliary programs Antechamber. The MD simulations were carried out with the ff99SB forcefield using TIP3P explicit water in a cubic box with an 8 Å distance around the complexes. The positive charges were subsequently neutralized with chloride ions. The Particle Mesh Ewald method [44] was used to calculate the long-range electrostatic interactions with a cut-off value of 12 Å, whereas the SHAKE algorithm [45] was applied to constrain all bonds involving hydrogen atoms. Initially, the solvated complexes were subjected to energy minimization carried out in two stages [46]. In the first stage, only ions and water molecules were relaxed by minimization over 2000 steps—i.e., an initial partial optimization of 1000 steps using the steepest descent algorithm followed by an optimization of 1000 steps using the conjugated gradient algorithm, by applying a restrained force of 500 kcal/mol to keep the solute kept fixed for 200 ps. Subsequently, another minimization was performed in which the whole system was relaxed by 5000 steps—i.e., a full optimization of 2500 steps using the steepest descent algorithm, followed by 2500 steps with the conjugated gradient algorithm. The minimized systems were then gradually heated up from 0 to 300 K with a weak harmonic restraint of 10 kcal mol^−1^ by keeping the complex fixed for 200 ps. This heating process was followed by equilibration for 2 ns in the *NVT* ensemble (*T* = 300 K), and then 50 ns MD simulations without any restrictions were run in the *NpT* ensemble at constant *T* = 300 K and *p* = 1 atm.

The stability of the protein–ligand complexes was determined by calculating the root mean square deviation (RMSD) of the complexes and ligands, the root mean square fluctuations (RMSF), and their radius of gyration (Rg). Furthermore, the molecular mechanics generalized born surface area (MM-GBSA) [47] binding free energies of the complexes were calculated using the MM-PBSA.py tool of Amber and by employing 100 snapshots taken from the last 10 ns of the MD trajectory. The entropy contribution (−*T*Δ*S*) of the binding free energies were calculated using normal mode analyses taking 100 snapshots from the last 10 ns of the MD trajectory.

## 3. Results and Discussion

### 3.1. Multi-Target QSAR Models

Before starting this section, we need to clarify that the first objective of this work was to obtain an mt-QSAR model with high predictive accuracy as well as mechanistic interpretability [48]. Undoubtedly, any linear mt-QSAR model developed with a limited number of descriptors serves both purposes. Nevertheless, such models often have drawbacks, as their predictivity is found to be less than that of non-linear models developed with a large number of features. On the other hand, some non-linear models have been reported in recent years with the most highly discriminating features available, based on differential Shannon entropy descriptors [27,49]. In fact, the latter models, if generated with high statistical quality, can also provide mechanistic interpretations of the targets. Therefore, our goal was to generate all three types of models, that is: (a) a 10-descriptor linear model, (b) a 10-descriptor non-linear model and (c) non-linear models with an unlimited number of features (i.e., with all descriptors). For each type of model, multiple feature selection techniques or ML tools were thus employed. The most predictive models obtained in this way are shown in Table 1.

**Figure 1:**
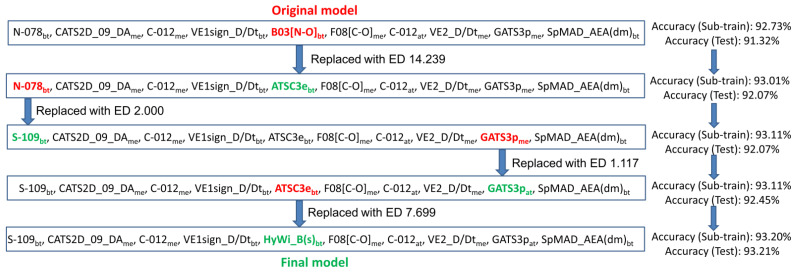
Flowchart showing the modification of the original FS-LDA model through PS3M refinement.

As can be seen, among the three different feature selection algorithms (FS, SFS and GA), the best linear classification model was generated with the FS-LDA scheme. Similarly, among the various ML tools, RF and GB afforded the most predictive non-linear models based on the limited number of descriptors (=10) and on all descriptors, respectively. Clearly, the GB-based non-linear model was found to be the most predictive one, judging by its accuracy values of 92.35%, 92.45% and 90.67% for the sub-training, test and external validation sets, respectively. As far as the predictive accuracy is concerned, this non-linear model is followed by the linear mt-QSAR model, leading to accuracy values of 92.73%, 91.32% and 89.61% for the sub-training, test and external validation sets, respectively. Therefore, even with a limited number of features, one can see that the statistical quality of the linear model is remarkably close to that of the non-linear model generated with an unlimited number of descriptors. This further implies that linear mt-QSAR modeling can lead to the most desirable model as it provides high predictive accuracy and, at the same time, projects the most significant structural features required for higher activity against MNK-1/2 under different experimental assay conditions. After confirming this, we used this linear mt-QSAR model for further processing using the ‘Post-selection similarity search-based modification’ technique (or PS3M) in order to check if any similar model may exist with a higher statistical quality. As depicted in Figure 1, starting from the originally developed linear model, four rounds of PS3M analyses were carried out and, in each step, the overall accuracy obtained from the sub-training and test sets was continuously improved. No further improvement was noticed after the fourth step, in which the accuracy of the sub-training set was dropped. Here, we need to reiterate that in this PS3M analysis, the statistical quality of the sub-training set was not compromised, and in the process, the test set is used as a calibration set so that the successive models are not over-fitted. The final model depicted accuracy values of 93.20% and 93.21% for the sub-training and test sets. Following this, we determined the predictive accuracy of the final model against the external validation set and an accuracy of 90.67% was obtained. Indeed, after performing the PS3M refinement, the quality of the final model improved for all sets and simultaneously, the overall performance of this model was found to be better than the GB-based non-linear model.

Table 2 shows a detailed description of the FS-LDA-based original model as well as of the refined PS3M model (henceforth referred as the final model), thus enabling us to compare these two models in all aspects. First, it is observed that the PS3M technique did not compromise the goodness-of-fit of the final model, judging from its Wilks λ value (=0.329). Furthermore, a closer look at the values of MCC, which is regarded as a robust parameter for validating the quality of classification model, allows us to infer that the final model is truly more predictive than the original model. Notice that the former yields highly satisfactory MCC values of 0.848, 0.855 and 0.785 for the sub-training, test and validation sets, respectively. The values of the descriptors, as well as the predicted activity of the original FS-LDA and final models, are provided in the Supplementary Materials (as Tables S3 and S4, respectively).

**Figure 2:**
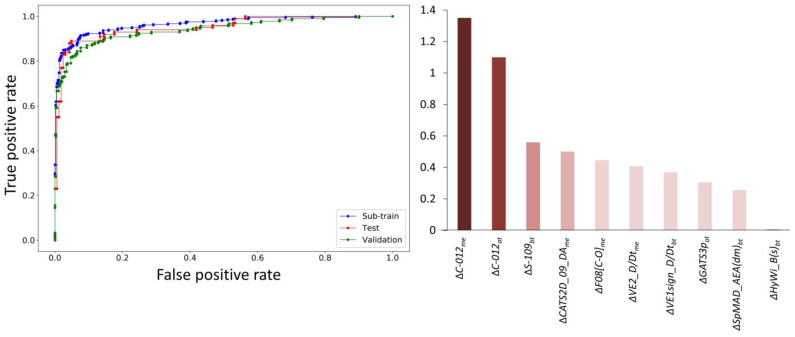
ROC plot of the final mt-QSAR model (**left**) and the absolute standardized coefficients of its descriptors (**right**).

Before finally accepting this model as the best linear mt-QSAR model, we had to confirm that the descriptors of the model are not highly correlated. This is ensured by the fact that the maximum correlation (R^2^) between any two descriptors of the model was found to be 0.66. Furthermore, we performed a *Y_c_*-randomization test that, with 100 runs, provided λ*_r_* and Accuracy*_r_* values of 0.993 and 64.97%, respectively. Therefore, it can be assumed that the generated model is unique and not developed by chance, as the scrambled parameters destabilize the goodness-of-fit of the models and at the same time, the accuracy regarding the sub-training set is reduced to a considerable extent. Furthermore, comparatively small $Acc_{rnd}$ values were obtained for each set of the final models, indicating that this model is capable of providing significant information at the maximal level of random accuracy [31,32]. The ROC plot of this final mt-QSAR model is shown in Figure 2, which clearly indicates a high predictive accuracy of the final model. More specifically, the AUROC scores obtained are 0.912, 0.923 and 0.878 for the sub-training, test and validation sets, respectively.

As previously mentioned, one of the main objectives of the current work is to understand the structural requirements of the compounds for them to have a higher affinity towards MNK-1/2 in different experimental assay conditions. Noticeably, all three experimental elements (i.e., *a_t_, m_e_* and *b_t_*) contributed to the development of the final mt-QSAR model, clearly indicating their influence. The significance of the descriptors appearing in the final mt-QSAR model can be inferred from their absolute standardized coefficients that are depicted in Figure 2. Interestingly, two of the most significant descriptors of the model (i.e., $\Delta (C-012{)}_{m_{e}}$ and $\Delta (C-012{)}_{a_{t}}$) are derived from the same descriptor C—012, which simply represents a molecular fragment of the type CR2 × 2 (where R is alkyl group and X is any heteroatom). A positive contribution to higher inhibitory potential is found when the modified descriptor derived from this fragment relates to the experimental element *m_e_* (measures of biological effects). However, a negative influence is noted when it is related to the other experimental element, i.e., *a_t_* (assay type). It infers that the CR2 × 2 fragment is definitely a significant contributor in determining the higher inhibitory potential of the compounds, but its role may vary on the basis of experimental conditions. Descriptor $\Delta (S-109{)}_{b_{t}}$ emerges as the third most influential descriptor of the model, which is based on another atom-centered fragment S—109 (presence or absence of an R–SO–R fragment) and the experimental element *b_t_* (biological target). It shows a positive correlation with the response variable, indicating that the fragment R–SO–R may have a favorable influence on the higher inhibitory potential of the compounds against these enzymes. Chemically advanced template search (CATS) descriptors are a very useful category of descriptors that attempt to explain the structural requirements on the basis of various pharmacophore groups, along with the topological distances that separate these [50]. For example, $\Delta (\mathrm{CATS}2D\_09{\_\mathrm{DA})}_{m_{e}}$, which shows a positive relationship with biological activity, is based on hydrogen bond donor and acceptor features located at a topological distance of nine. The fifth most important descriptor of the model is $\Delta (F08[C-{O])}_{m_{e}}$, which is an atom-pair descriptor accounting for the frequency of C–O at a topological distance of eight. Both these two latter descriptors are dependent on the experimental element *m_e_*. However,$\Delta (F08[C-{O])}_{m_{e}}$ showed a negative relation with the higher inhibitory potential. The remaining five descriptors of the models are graph-based topological descriptors. Two of them, i.e., descriptors ${\Delta (\mathrm{VE}2\_D/Dt)}_{m_{e}}$ and ${\Delta (\mathrm{VE}1\mathrm{sign}\_D/Dt)}_{b_{t}}$, are 2D matrix-based descriptors derived from the distance/detour matrix, standing namely for the average coefficient of the last eigenvector (VE2_D/Dt) and for the coefficient sum of the last eigenvector (VE1sign_D/Dt). The presence of such descriptors in the model indicates that topological distributions of mass, charge and lipophilicity in the compounds contribute significantly to determining the inhibitory potential of the compounds [51]. The importance of topological distributions of polarizability and dipole moments is reflected by descriptors such as ${\Delta (\mathrm{GATS}3p)}_{a_{t}}$ and ${\Delta (\mathrm{SpMAD}\_\mathrm{AEA}(\mathrm{dm}))}_{b_{t}}$. The former GATS3p is a 2D-autocorrelation descriptor that stands for the Geary autocorrelation of lag 3 weighted by polarizability. Similarly, SpMAD_AEA(dm) is a graph-based edge-adjacency index that stands for the spectral mean absolute deviation from the augmented edge adjacency matrix weighted by the dipole moment. Finally, the least significant descriptor ${\Delta (\mathrm{HyWi}\_B(s))}_{b_{t}}$ is also another 2D matrix-based descriptor that represents the hyper-Wiener-like index (log function) from the Burden matrix, weighted by the intrinsic state of the atoms [52].

Table 3 shows the results of the technique formerly termed as ′condition-wise prediction′ [18]. Application of this technique allows one to understand how the mt-QSAR model performs against various experimental conditions, resulting from the combination of the three experimental elements present in the modeling dataset. As can be seen, seven different experimental conditions are found in the modeling dataset. As far as the external predictivity is concerned, the model afforded high accuracy values against most of these experimental conditions. The lowest predictivity was however obtained for Condition 5 (*m_e_*: *K_i_*, *a_t_*: B, *b_t_*: MNK-2). Nevertheless, for this condition too an overall accuracy greater than 80% was achieved.

### 3.2. Virtual Screening of Potential Hits

Whenever a predictive model is developed it offers an enormous opportunity for screening chemical libraries to find potential lead molecules [53]. Indeed, large databases may be used for screening and, at the same time, some researchers may prefer to screen their in-house databases that are not available in the public domain. Therefore, it is imperative that the developed models are easily accessible to users, so that they may easily apply these for screening of any database of interest. In order to address this issue, we used publicly available in-house tools for developing the mt-QSAR models. Furthermore, considering the fact that model development may itself be a time-consuming task for new or users unfamiliar with computational chemistry applications, we implemented another FLASK-based application that can be run on local machines. This application allows the users to quickly predict their own database of interest using the most predictive mt-QSAR model, and is available in the Github repository (https://github.com/ncordeirfcup/MNK_model/tree/master).

Additionally, here we performed a virtual screening using the Asinex kinase library (http://www.asinex.com/focus_kinases/, accessed on 4 July 2021), which is a focused library of potential kinase inhibitors containing 6538 small molecules. Virtual screening endeavors carried out with any mt-QSAR model should also ensure that the most potential virtual hits are predicted as potential inhibitors over most of the targeted combinations of elements considered. In the current work, seven such combinations were found (listed in Table 3) and therefore, the virtual screening dataset contained 45,766 (=7 × 6538) compounds, the descriptors of which were calculated in the same manner as the descriptors of the modeling dataset. Following this, the inhibitory potential of these compounds was evaluated using the most predictive mt-QSAR model developed in this work. In search of pan-inhibitors, we looked for those compounds that show activity against both isoforms. After analyzing the results, only 20 such compounds were found to display activity against at least 4 out of 7 combinations of experimental elements, and none of them were found to be a structural outlier of the model. It is important to mention that Box–Jenkins-based moving average techniques are not only suitable for design of pan-inhibitors. These may also be utilized in the selection of compounds intended to be designed as isoform-specific inhibitors. For example, in order to search MNK-2 specific inhibitors, we need to first select those hits that are predicted to have activity against MNK-2, but where no activity is found against MNK-1. At the same time, these inhibitors that are required to be active under a maximum number of experimental conditions.

**Figure 3:**
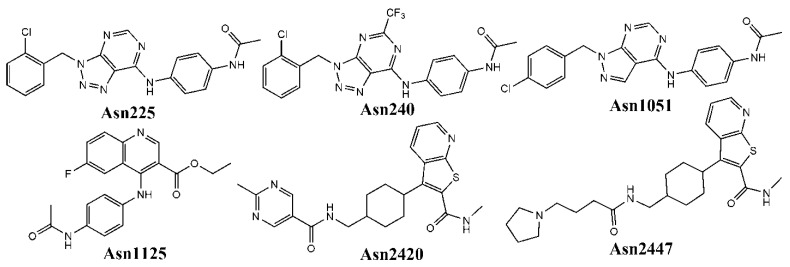
Molecular structures of the six virtual hits chosen after the similarity search analysis.

For post-screening filtering, we applied a similarity search-based analysis to select virtual hits that possess maximum structural similarities with the already reported MNK-1/2 inhibitors. To do so, we first prepared a dataset of reported MNK-1 and MNK-2 inhibitors from the Binding Database (https://www.bindingdb.org/bind/index.jsp, accessed on 7 July 2021), another web-accessible database of measured binding affinities that is focused mainly on the interactions of protein drug targets with small molecules. In so doing, we managed to collect 1983 compounds from this database with reported inhibitory potential (IC_50_, *K_d_*, *K_i_*) of less than 5000 nM against these two drug targets. Such compounds (see Table S5) served as a target dataset whereas 20 virtual hits were used as a query dataset. The similarity searching was done with our in-house tool named SIMSEARCH, which first calculates the ECFP4 fingerprints of all queries as well as the target dataset compounds, and then, computes the Tanimoto similarity between each target and query dataset compound. We took a maximum cut-off value of 0.3 for the Tanimoto similarity to identify those query dataset compounds that show a high structural similarity with the target dataset compounds. Table 4 depicts the number of matches found in this similarity search process. It can be seen that only 14 virtual hits were found to match with at least one target dataset compound with a structural similarity greater than 0.3. In the current work however, we selected the top six query virtual hits that were found to match more than five reported MNK-1/2 inhibitors—these inhibitors are Asn1051, Asn0225, Asn1125, Asn2420, Asn0240 and Asn2447 (see Figure 3).

The rationale behind the selection of a high average inhibitory activity cut-off value (cf. Table 4) stems from the fact that similarity searching just provides a hint about how much confidence one may confer on the selected virtual hits from the context of the structures of already reported potent inhibitors. Therefore, even though a query like Asn1051 shows an average MNK-1/2 inhibitory activity (i.e., 1085.78 nM) higher than the selected cut-off value used for developing our QSAR models (i.e., 100 nM for IC_50_ and 300 nM for *K_i_* and *K_d_*), this query compound or virtual hit may certainly have much improved the inhibitory potential towards these enzymes, as its structure is similar but not exactly the same as the query compounds.

**Figure 4:**
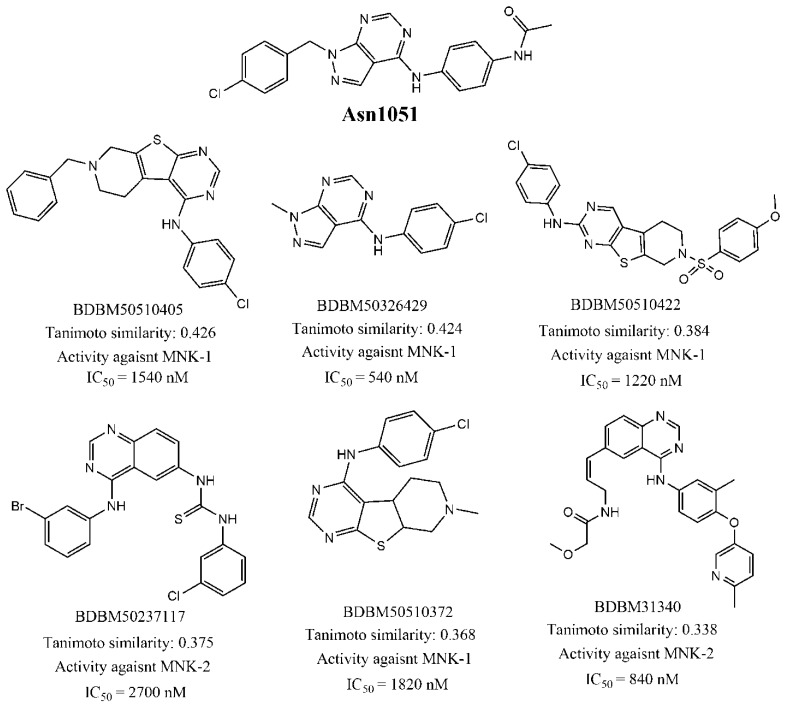
Six target dataset matches of Asn1051, with reported MNK-1/2 activity in the Binding Database, found by the similarity search analysis.

Let us now demonstrate how the similarity search worked by selecting the Asn1051 compound as a reference. It is observed in Table 4 that this compound matched with 45 target dataset compounds with an average Tanimoto similarity of 0.33 and an average MNK-1/2 inhibitory activity of 1085 nM. Now, Figure 4 displays six such target dataset compounds, along with their Binding Database names, their Tanimoto similarity with Asn1051, and their experimental activity against MNK-1 or MNK-2. The similarity of Asn1051 with the structures of the latter compounds (especially with BDBM50326429) is easily recognizable. Definitely, the structural similarity between Asn1051 and all its matches in the target dataset clearly suggests that the former may indeed act as a potential MNK-1/2 inhibitor.

Subsequently, the top six virtual hits taken (i.e., Asn1051, Asn225, Asn1125, Asn2420, Asn240 and Asn2447) were analyzed with the SwissADME webserver (http://www.swissadme.ch/index.php, accessed on 10 July 2021) [54] to predict in silico drug-likeness, ADME profiles and synthetic accessibility of each of these. Looking at the summary of these predictions shown in Table 5, one can observe that all these hit compounds have satisfactory ADME profiles. In addition, none of these was found to violate the Lipinski’s rule of five [55] or the Vebers rule [56], which are used for checking drug-likeness. What is more, the low synthetic accessibility scores obtained for these compounds indicate that they are easily synthesizable.

**Figure 5:**
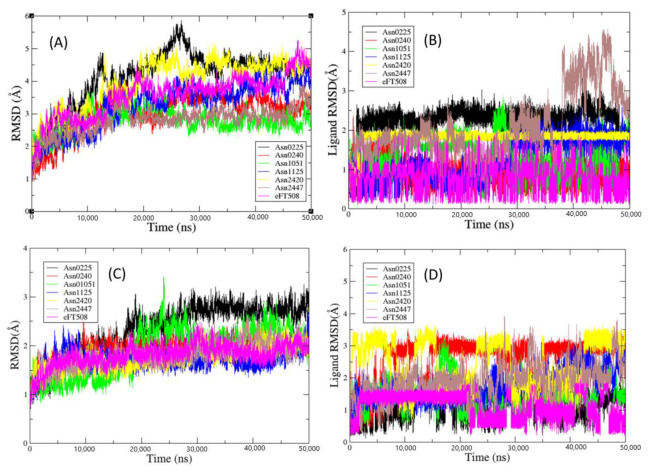
(**A**) RMSD plot of MNK-2 protein complexes and (**B**) their associated ligands. (**C**) RMSD plot of MNK-1 protein complexes and (**D**) their associated ligands.

Nonetheless, we finally employed the results of the MD simulations for selecting the most promising virtual hits. To do so, these six virtual hits were firstly docked against the X-ray crystal structures of MNK-1 and MNK-2. Then, we carried out 50 ns of MD simulations of the obtained docked protein–ligand complexes. For comparative purposes, the docked poses obtained for the well-known MNK-1/2 inhibitor eFT508 were employed as positive controls and also subjected to similar MD simulations. The dynamic stability of all these complexes was checked by inspection of the RMSD, RMSF and RG plots of the protein–ligand complexes, as well as of the RMSD plots of their ligands, gathered from the MD runs. The RMSD plots obtained from protein complexes and ligands are presented in Figure 5, whereas the RMSF and RG plots are provided in the Supplementary Materials (Figures S1 and S2).

Such plots suggest that all complexes and ligands reach a properly dynamic stability during the simulations. Since our main goal is to estimate the binding affinity of the selected hits along with that of the positive control against the targeted enzyme isoforms, Table 6 shows the calculated corresponding MM-GBSA binding free energies (Δ*G*_bind_ in kcal/mol). As can be seen, save for Asn1125 and Asn2447, all other virtual hits depict theoretical binding free energies less than −35 kcal/mol and, when compared to the positive control eFT508, display a satisfactory binding affinity towards the two enzyme isoforms. The maximum average binding affinity pertains to Asn0225, whereas Asn1051 shows the maximum consistency in the binding free energies against the two MNK isoforms. Overall, the MD results thus indicate that the most promising MNK-1/2 virtual hits are the following compounds: Asn0225, Asn0240, Asn1051 and Asn2420.

## 4. Conclusions

In silico based multi-target (or multi-tasking) QSAR modeling is a powerful tool for understanding the structural requirements for higher inhibitory potential of a large set of compounds against multiple biological targets under a variety of experimental assay conditions. Additionally, it is especially suited for a fast identification of potential virtual hits against the same biological targets. As such, this work aimed to develop mt-QSAR models targeting the inhibitors of two very important enzyme isoforms, namely: MNK-1 and MNK-2. To do so, we collected a large dataset comprising 1892 compounds with activity against these two biological targets under various experimental assay conditions and then, we applied multiple feature selection algorithms, ML tools, model development and validation strategies to establish predictive and accurate mt-QSAR models. Interestingly, we found that the performance of the linear model developed with a limited number of descriptors performed better than all non-linear models as far as predictive accuracy is concerned. Besides this, that linear model provided us with information regarding the needed structural requirements for higher inhibitory potential towards MNK-1/2. It was found that some molecular fragments, such as CR2X2 and R–SO–R, as well as hydrogen bond donor/acceptor groups and C–O pairs at certain topological distances, play significant roles in ascertaining the compounds’ inhibitory potential against MNK-1/2. Atomic polarizabilities, dipole moments and intrinsic states also appear to play key contributions in their likely inhibition. It was further noticed that the linear model performed in a satisfactory manner, irrespectively of the experimental conditions. Additionally, all QSAR models were set up by employing software, tools and webservers that are publicly accessible in order to allow their easy reproduction. Furthermore, realizing that our highly predictive linear model (average accuracy >90%) may be used by other researchers for screening chemical libraries (both in-house and commercial), we made the screening facility using this model more easily accessible by implementing a FLASK-based application available in the Github repository (https://github.com/ncordeirfcup/MNK_model/tree/master). At the same time, we also performed a virtual screening with a focused kinase library to identify some potential virtual hits. Even though that screening mainly relied on predictions done with the most predictive mt-QSAR model, we also resorted to similarity search analysis as well as molecular modeling techniques, including MD simulations to improve confidence towards our finally proposed six virtual hits. To sum up, all the information obtained in this work can guide the future discovery of novel MNK-1/2 inhibitors as potential therapeutic agents.

## Figures and Tables

**Table 1:** Statistical results of the mt-QSAR models generated with different model building strategies.

Parameters	Linear Model (Ten-Descriptor; FS-LDA)			Non-Linear Model (Ten-Descriptor; RF)			Non-Linear Model (All Descriptor; GB)		
	Sub-Train	Test	Validation	Sub-Train	Test	Validation	Sub-Train	Test	Validation
TP	308	88	147	314	81	148	318	87	150
TN	674	154	362	653	152	361	660	158	365
FP	20	11	20	41	13	21	34	7	17
FN	57	12	39	51	19	38	47	13	36
Sensitivity	97.12	93.33	94.76	86.03	92.12	94.51	87.12	95.76	95.55
Specificity	84.38	88.00	79.03	94.09	81.00	79.57	95.10	87.00	80.64
Accuracy	92.73	91.32	89.61	91.31	87.92	89.61	92.35	92.45	90.67
F1-score	88.89	88.44	83.29	87.22	83.50	83.38	88.70	89.69	84.98
MCC	0.838	0.815	0.76	na	0.741	0.760	na	0.838	0.785
AUROC	0.907	0.907	0.869	na	0.866	0.870	na	0.914	0.881

**Table 2:** Comparison between the original FS-LDA model and the final LDA model produced by PS3M refinement.

Model	Equation	Sub-Training	Test	Validation
Original (FS-LDA)	$\begin{array}{l} \mathrm{IA}c_{j}=+4.003+5.035\;\Delta (N-078{)}_{b_{t}}+1.633\;\Delta (\mathrm{CATS}2D\_09{\_\mathrm{DA})}_{m_{e}}\\ \;\;\;\;\;+20.515\;\Delta (C-012{)}_{m_{e}}+6.210\;{\Delta (\mathrm{VE}1\mathrm{sign}\_D/Dt)}_{b_{t}}+1.608\;\Delta (B03[N-{O])}_{b_{t}}\\ \;\;\;\;\;-0.600\;\Delta (F08[C-{O])}_{m_{e}}-17.842\;\Delta (C-012{)}_{a_{t}}-45.641\;\Delta (\mathrm{VE}2\_D/Dt\;{)}_{m_{e}}\\ \;\;\;\;\;-7.661\;{\Delta (\mathrm{GATS}3p)}_{m_{e}}+16.232\;{\Delta (\mathrm{SpMAD}\_\mathrm{AEA}(\mathrm{dm}))}_{b_{t}} \end{array}$ Wilks λ = 0.319, *F* = 224.16, *p* < 10^−16^	TP = 308 TN = 674 FP = 20 FN = 57 Sn = 97.12 Sp = 84.38 Acc = 92.73 F1 = 88.89 MCC = 0.838 $Acc_{rnd}=55.91$	TP = 88 TN = 154 FP = 11 FN = 12 Sn = 93.33 Sp = 88.00 Acc = 91.32 F1 = 88.44 MCC = 0.815 $Acc_{rnd}=53.10$	TP = 147 TN = 362 FP = 20 FN = 39 Sn = 94.76 Sp = 79.03 Acc = 89.61 F1 = 83.29 MCC = 0.760 $Acc_{rnd}=57.11$
Final (PS3M)	$\begin{array}{l} \mathrm{IA}c_{j}=+4.000+1.532\;\Delta (\mathrm{CATS}2D\_09{\_\mathrm{DA})}_{m_{e}}+21.079\;\Delta (C-012{)}_{m_{e}}\\ \;\;\;\;\;+6.037\;{\Delta (\mathrm{VE}1\mathrm{sign}\_D/Dt)}_{b_{t}}-0.546\;\Delta (F08[C-{O])}_{m_{e}}-17.982\;\Delta (C-012{)}_{a_{t}}\\ \;\;\;\;\;-52.531\;\Delta (\mathrm{VE}2\_D/Dt\;{)}_{m_{e}}+16.415\;{\Delta (\mathrm{SpMAD}\_\mathrm{AEA}(\mathrm{dm}))}_{b_{t}}+5.531\;\Delta (S-109{)}_{b_{t}}\\ \;\;\;\;\;-7.509\;{\Delta (\mathrm{GATS}3p)}_{a_{t}}-0.050\;{\Delta (\mathrm{HyWi}\_B(s))}_{b_{t}} \end{array}$ Wilks λ = 0.329, *F* = 212.86, *p* < 10^−16^	TP = 310 TN = 677 FP = 17 FN = 55 Sn = 97.55 Sp = 84.93 Acc = 93.20 F1 = 89.59 MCC = 0.848 $Acc_{rnd}=55.94$	TP = 89 TN = 158 FP = 7 FN = 11 Sn = 95.76 Sp = 89.00 Acc = 93.20 F1 = 90.82 MCC = 0.855 $Acc_{rnd}=53.38$	TP = 148 TN = 367 FP = 15 FN = 38 Sn = 96.07 Sp = 79.57 Acc = 90.67 F1 = 8 4.81 MCC = 0.785 $Acc_{rnd}=57.35$

**Table 3:** Results of the condition-wise prediction for the best mt-QSAR model.

Condition	*m_e_*	*a_t_*	*b_t_*	Test Set		External Validation Set	
				#Instances	%Accuracy	#Instances	%Accuracy
1	IC_50_	B	MNK-2	189	88.36	107	92.52
2	IC_50_	B	MNK-1	104	88.46	40	92.50
3	*K_d_*	B	MNK-2	20	95.00	11	90.91
4	*K_d_*	B	MNK-1	31	96.77	9	100.00
5	*K_i_*	B	MNK-2	19	84.21	1	0.00
6	*K_i_*	B	MNK-1	15	93.33	5	100.00
7	*K_i_*	F	MNK-2	190	93.16	92	94.57

**Table 4:** Results of the similarity search analysis ***^a^***.

Query Compounds	Number of Matches	Average MNK-1/2 Activity *^b^*	Average Similarity
Asn1051	45	1085.78	0.33
Asn0225	30	1218.10	0.32
Asn1125	14	646.57	0.32
Asn2420	14	36.36	0.32
Asn0240	12	608.00	0.32
Asn2447	6	22.50	0.32
Asn0252	4	2100.00	0.33
Asn2416	3	35.00	0.32
Asn2471	3	45.00	0.31
Asn2459	2	36.50	0.31
Asn2466	2	49.00	0.32
Asn4780	2	1032.00	0.33
Asn2422	1	46.00	0.31
Asn2458	1	46.00	0.32

***^a^*** All matches in the target dataset show a Tanimoto similarity value greater than 0.3 with the query compound. ***^b^*** In this calculation, if the inhibitory potential of a compound is expressed as < 100 nM, it was considered equal to 100 nM.

**Table 5:** ADME, drug-likeness and synthetic accessibility of the virtual hit compounds as predicted by the SwissADME webserver.

Compound	ESOL *^a^* Class	GI *^b^* Absorption	BBB *^c^* Permeant	*p*-gp *^d^* Substrate	Lipinski #Violations	Veber #Violations	Synthetic Accessibility
Asn0225	Moderate	High	No	No	0	0	3.06
Asn0240	Moderate	High	No	No	0	0	3.28
Asn1051	Moderate	High	No	No	0	0	2.77
Asn1125	Moderate	High	No	No	0	0	2.67
Asn2420	Moderate	High	No	Yes	0	0	4.18
Asn2447	Moderate	High	No	Yes	0	0	4.44

***^a^*** Estimated aqueous solubility. ***^b^*** GI: Gastrointestinal. ***^c^*** BBB: Blood–Brain Barrier. ***^d^*** *p*-gp: *p*-glycoprotein.

**Table 6:** Calculated binding free energies (Δ*G*_bind_ in kcal/mol) for the MNK-1 and MNK-2 bound ligands.

Query Compounds	MNK-1	MNK-2
Asn1051	−40.20	−37.21
Asn0225	−52.97	−35.21
Asn1125	−32.32	−37.45
Asn2420	−38.38	−48.94
Asn0240	−32.33	−42.28
Asn2447	−31.88	−29.71
eFT508	−36.41	−44.82

## Data Availability

Further details about the data presented in this study are available on request from the corresponding authors.

## Supplementary Materials

The following are available online at https://www.mdpi.com/article/10.3390/biom11111670/s1, Figure S1: RMSF plot of MNK-1 proteins (left) and radius of gyration plot of MNK-1 protein complexes (right). Figure S2: RMSF plot of MNK-2 proteins (left) and radius of gyration plot of MNK-2 protein complexes (right). Table S1: Details of the dataset used for setting up the mt-QSAR models against the MNKs. Table S2: The architecture of the deep neural network (DNN) model used in the current work and in a previous report of Sosnin et al., Table S3: The values of the descriptors, the observed and the predicted activity of the original FS-LDA. Table S4. The values of the descriptors, the observed and the predicted activity of the final FS-LDA. Table S5: Details of the Binding dataset compounds used for similarity search analyses. DNN_MNK_file1.ipynb and DNN_MNK_file2.ipynb: Jupyter notebook files for DNN model development.
