# Supplementary material for: Multi-Target In Silico Prediction of Inhibitors for Mitogen-Activated Protein Kinase-Interacting Kinases
Source: Biomolecules. 2021 Nov 10;11(11):1670. doi: 10.3390/biom11111670 (PMC8615736; doi:10.3390/biom11111670)
Supplement: Supplementary file 1 [file biomolecules-11-01670-s001.zip › Table S2.pdf]

Multi-Target in silico prediction of Inhibitors for Mitogen Activated Protein Kinase-Interacting Kinases

Amit Kumar Halder [1,2*], and M. Natália D. S. Cordeiro [1,*]

[1] LAQV-REQUIMTE/Faculty of Sciences, University of Porto, 4169-007 Porto, Portugal
[2] Dr. B. C. Roy College of Pharmacy and Allied Health Sciences, Dr. Meghnad Saha Sarani, Bidhannagar, Durgapur 713212, West Bengal, India
* Correspondence: amit.halder@fc.up.pt (A.K.H.); ncordeir@fc.up.pt (M.N.D.S.C.)

Table S2. The architecture of Deep Neural Network (DNN) model used in the current work and in a previous report of Sosnin et al. [1].

Hidden Layer	Neurons	Batch Normalization	Dropout ratio
1	512	Yes	0.50
2	256	Yes	0.50
3	128	Yes	0.50
4	64	Yes	0.50
5	32	Yes	0.25
6	32	No	0.10

References

1. Sosnin, S.; Karlov, D.; Tetko, I.V.; Fedorov, M.V. Comparative Study of Multitask Toxicity Modeling on a Broad Chemical Space. *J. Chem. Inf. Model.* **2019**, *59*, 1062-1072.
